# General Principles for the Safe Performance, Training, and Adoption of Ablation Techniques for Benign Thyroid Nodules: An American Thyroid Association Statement

**DOI:** 10.1089/thy.2023.0281

**Published:** 2023-10-13

**Authors:** Catherine F. Sinclair, Jung Hwan Baek, Kathleen E. Hands, Steven P. Hodak, Timothy C. Huber, Iram Hussain, Brian Hung-Hin Lang, Julia E. Noel, Maria Papaleontiou, Kepal N. Patel, Gilles Russ, Jonathon Russell, Stefano Spiezia, Jennifer H. Kuo

**Affiliations:** ^1^ Icahn School of Medicine, New York, New York, USA.; ^2^ Department of Otolaryngology, Monash University, Melbourne, Australia.; ^3^ Department of Radiology and Research Institute of Radiology, University of Ulsan College of Medicine, Asan Medical Center, Seoul, Korea.; ^4^ Thyroid Center of South Texas, San Antonio, Texas, USA.; ^5^ Department of Medicine, NYU Grossman School of Medicine, New York, New York, USA.; ^6^ Department of Interventional Radiology, Oregon Health and Science University, Portland, Oregon, USA.; ^7^ Division of Endocrinology, Department of Internal Medicine, University of Texas Southwestern Medical Center, Dallas, Texas, USA.; ^8^ Department of Surgery, University of Hong Kong, Queen Mary Hospital, Pokfulam, Hong Kong.; ^9^ Department of Otolaryngology Head & Neck Surgery, Stanford University School of Medicine, Stanford, California, USA.; ^10^ Division of Metabolism, Endocrinology and Diabetes, University of Michigan, Ann Arbor, Michigan, USA.; ^11^ Division of Endocrine Surgery, Department of Surgery, New York University Langone Health, Bethesda, Maryland, USA.; ^12^ Thyroid Diseases and Endocrine Tumors Department, Pitié-Salpêtrière Hospital, Paris, France.; ^13^ Institute of Cancer IUC, Clinical Research Group Thyroid Tumors No. 16, Sorbonne University, Paris, France.; ^14^ Department of Otolaryngology-Head and Neck Surgery, Johns Hopkins School of Medicine, Baltimore, Maryland, USA.; ^15^ Endocrine and Ultrasound Guided Surgery Operative Unit, Ospedale del Mare, ASLNA1Centro, Naples, Italy.; ^16^ Section of Endocrine Surgery, Department of Surgery, Columbia University, New York, New York, USA.

**Keywords:** ablation, benign thyroid nodule, ultrasound, thyroid nodules

## Abstract

**Background:**

The primary goal of this interdisciplinary consensus statement is to provide a framework for the safe adoption and implementation of ablation technologies for benign thyroid nodules.

**Summary:**

This consensus statement is organized around three key themes: (1) safety of ablation techniques and their implementation, (2) optimal skillset criteria for proceduralists performing ablative procedures, and (3) defining expectations of success for this treatment option given its unique risks and benefits. Ablation safety considerations in pre-procedural, peri-procedural, and post-procedural settings are discussed, including clinical factors related to patient selection and counseling, anesthetic and technical considerations to optimize patient safety, peri-procedural risk mitigation strategies, post-procedural complication management, and safe follow-up practices. Prior training, knowledge, and steps that should be considered by any physician who desires to incorporate thyroid nodule ablation into their practice are defined and discussed. Examples of successful clinical practice implementation models of this emerging technology are provided.

**Conclusions:**

Thyroid ablative procedures provide valid alternative treatment strategies to conventional surgical management for a subset of patients with symptomatic benign thyroid nodules. Careful patient and nodule selection are critical to the success of these procedures as is extensive pre-procedural patient counseling. Although these emerging technologies hold great promise, they are not without risk and require the development of a unique skillset and environment for optimal, safe performance and consistent outcomes.

## INTRODUCTION

Thermal and chemical ablation refers to a group of versatile, non-surgical techniques that are used to treat benign thyroid nodules. In North America, chemical ablation techniques have been utilized for decades; however, thermal techniques have only recently been introduced. Ablative techniques are minimally invasive compared with surgery; however, there is a potential for complications and morbidity if the procedures are performed incorrectly or by inexperienced practitioners.

Many international case series and consensus statements have been published in the past decade while evaluating and summarizing the indications, contra-indications, and outcomes of thermal and chemical ablation techniques for benign thyroid nodules.^1–14^ However, there are no documents to date in the United States focusing primarily on the safe adoption and implementation of ablation techniques, including learning curve considerations and necessary pre-procedural skillsets.

The objective of this American Thyroid Association (ATA) Statement with endorsement by the American Association of Endocrine Surgeons (AAES), American Academy of Otolaryngology Head and Neck Surgery (AAO-HNS), American Head and Neck Society (AHNS), Society of Interventional Radiology (SIR), Latin-American Thyroid Society (LATS), Asia and Oceania Thyroid Association (AOTA), and the Asia Pacific Society of Thyroid Surgery (APTS) is to provide a framework for the safe adoption and implementation of thermal and chemical ablative technologies for benign thyroid nodules in the United States by (1) defining and discussing safety considerations in pre-procedural, peri-procedural, and post-procedural settings; (2) recognizing that although these emerging technologies hold promise, they are not without risk and require the development of a unique skillset for optimal, safe performance; and (3) defining the training, prior knowledge, and steps that should be considered by any physician who desires to incorporate thyroid nodule ablation into their practice.

This statement is targeted at surgeons, endocrinologists, and interventional radiologists who either currently perform or intend to perform thermal and/or chemical ablation of thyroid nodules, and other medical practitioners who manage patients with thyroid nodules, including those who intend to undergo or have undergone thyroid nodule ablation.

The multi-disciplinary author panel comprises clinicians with extensive knowledge of thyroid ablation techniques who have worked together to develop a safety framework for ablative procedures in the United States, from pre-procedural assessment to peri-procedural considerations and post-procedural follow-up.

## METHODOLOGY

Selection of the multidisciplinary author panel was overseen by the Board of Directors of the ATA with adherence to diversity and inclusion measures. The final author panel comprised clinicians of diverse clinical specialty and background who have extensive experience in ablative technologies, with representatives from endocrine surgery, head and neck surgery, endocrinology, and interventional radiology.

In line with and echoing some of the official policy of the ATA, our recommendations do not replace sound clinical judgment, capture all nuances likely to be present in any patient, nor supplant patient directives. Similarly, specific outcomes on following the recommendations put forward in this statement are not guaranteed.

We recommend that treatment decisions be based on independent judgments of health care providers carefully considering each patient's individual circumstances and their goals of care (established at the outset and revisited frequently) as well as feasibility considerations (including regional access to specific health care resources). We expect that those who use this guideline will do so as an aid in, not a replacement for, sound and thoughtful clinical decision making, with full consideration of each patient's individuality in terms of history and physical traits, comorbidities, functional status, and goals of care.

### Phase 1

An initial outline of the statement in the form of a draft paper was circulated. The author panel reviewed the outline, and a videoconference was held to discuss the document. Panelists gave feedback, and the outline was modified accordingly before the layout was finalized. Each author was assigned a section of the paper based on their particular skillset or interest.

Literature searches were performed by the authors of each proposed section to identify relevant articles in multiple databases between 1990 and February 2023, including the Cochrane Library, EMBASE, PubMed, Infobase, the Cochrane Central Register of Controlled Trials (CENTRAL), and MEDLINE. Search terms included ablation, thermal ablation, chemical ablation, ethanol ablation, radiofrequency ablation, laser ablation, microwave ablation, high-intensity focused ultrasound (HIFU), and thyroid nodules.

Articles acquired from this search strategy combined with expert opinion were utilized to write the sections. After each author had submitted their contribution, the final article was collated by the lead authors (C.F.S., J.H.K.) and the full draft manuscript sent to the entire panel for commentary. The manuscript was modified in alignment with the comments received and a second draft recirculated for approval by the author panel.

### Phase 2

Section authors proposed draft Statement Recommendations in alignment with the Institute of Medicine's principles of health care quality, with the goal of addressing safety, access, appropriateness, efficiency, effectiveness, and patient centeredness. The entire author panel reviewed these statements, and modifications were made based on the feedback received by the lead authors of the statement (C.F.S., J.H.K.).

The modified Delphi method, a previously described and established method to systematically establish consensus, was utilized to determine which draft recommendations achieved consensus, near-consensus, or non-consensus.^15^ The panel of statements were delivered to the group electronically, via separate entry forms, so as to blind panelists to the responses of other members of the group.

The statements were circulated to all authors, and a vote was taken on each statement using a 1–5 Likert scale. Respondents were also given the opportunity to comment and provide feedback on each statement. Responses to the statement panel were collected electronically, and a dataset stripped of panel member names was created. These masked results were analyzed using a Likert scale with numerical interpretations of 1–5, utilizing the following anchor points: 1 (strongly disagree) and 5 (strongly agree). Statements were defined as achieving consensus if >70% of respondents agreed/strongly agreed with a statement after two rounds of the survey.

The completed manuscript was distributed to members of the author panel for final discussion, review, and approval.

### Phase 3

A single-phase comment period was conducted. Invitations were made to members of the ATA and endorsing societies. More than 160 comments were generated, including suggestions and technical considerations. Each comment was individually considered, and the vast majority were implemented in some capacity into the final document.

### Management of potential competing interests

To minimize to the greatest extent possible any potential influences of conflicts of interest on the opinions herein expressed, no personal financial conflicts of interest were permitted of the Task Force chairs. At inception of formation of the statement writing group, competing interests of the authors were reviewed by the Guideline Chairs, ATA Guidelines and Statements Committee, and Board of Directors of the ATA.

Given that the focus of the Statement is on principles of procedural safety, and not intended to be a guideline that recommends procedural indications or outcomes, all declared potential financial competing interests were deemed non-exclusionary for participation in the writing group. No potential competing interests were acquired during the development of the guidelines. All identified potential financial competing interests are declared in the Supplementary Data S1. No external funding from industry was received by the ATA or by authors for statement development.

## DISCUSSION

An overview of the discussion points of this safety statement is provided in Table 1.

**Table 1.** General Overview of Safety Statement

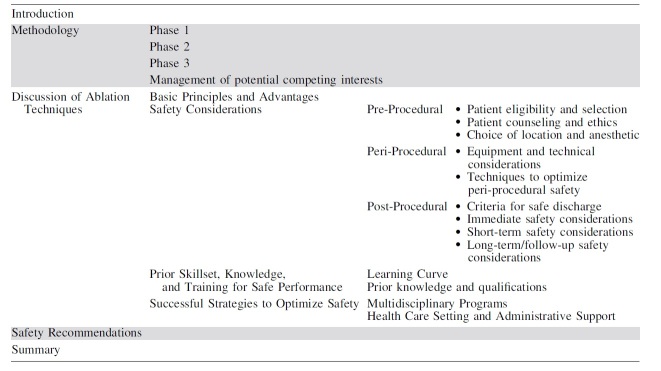

### Ablation techniques: basic principles and advantages

Chemical ablation, most commonly with dehydrated ethanol, causes both coagulative necrosis (via cell dehydration) and ischemic necrosis (by small vessel thrombus formation).^16^ Thermal ablation (TA) causes tissue coagulative necrosis by raising cell temperature to over 50–60°C (120–140°F)^17^, and the various TA techniques are differentiated by their method of generating heat. Basic principles for the different ablative technologies are as follows.

#### Ethanol ablation

Ethanol ablation (EA) involves injecting 95–99% dehydrated alcohol into a target nodule (cystic or mixed cystic-solid). Ethanol can be left *in situ* or aspirated out after a certain dwell time. Unpredictable diffusion within solid nodules makes this technique more suitable for cystic nodules.^11^

#### TA techniques

(1) Radiofrequency ablation (RFA): RFA uses an electrode to generate a high frequency alternating current (200–1200 kHz) that agitates the ions in the tissue, resulting in ionic excitation (frictional or resistive) causing heat production. This heat is then transmitted via conduction to adjacent tissue, causing further ablation.^1–6^
(2) Laser ablation (LA): LA utilizes single or multiple optical fibers to deliver a focused beam of light energy to the tissue, which results in photons transferring kinetic energy to the atoms, thus generating heat (7). The typical configuration used is a Nd:YAG Diode laser with emission wavelength of 1064 nm (near infrared wavelength), along with simulation software.^7,8^
(3) Microwave ablation (MWA): MWA involves creation of an electromagnetic field using microwaves of frequencies between 900 and 2500 MHz, which are emitted from a needle-like antenna. This leads to oscillation of polar water molecules and subsequent generation of frictional heat.^9^
(4) HIFU: HIFU focuses high intensity ultrasound (US) waves at a specific target location with resultant vibration of atoms, leading to the generation of frictional heat at the focal point.^10^ As the temperature rises, interstitial fluid boils and form microbubbles within the tissue, and expansion followed by collapse of these microbubbles induces hemorrhage within surrounding cells, through a process called cavitation.^10^ The result is both thermal and mechanical injury.

In most studies on ablation to date, TA complication rates are low and volume reductions are >50% at 12 months post-procedure, as summarized in Appendix Table A1.

### Ablation techniques: safety considerations

#### Pre-procedural

##### Patient eligibility and selection

###### Thermal ablation

TA for benign nodules is most appropriate for patients with compressive and/or cosmetic complaints that can be clearly attributed to a single or dominant nodule. Patients with autonomously functioning thyroid nodules (AFTNs) causing subclinical or overt hyperthyroidism can also be successfully treated with ablative techniques. Because ablation does not allow for definitive histologic analysis of the nodule, a firm conviction of benignity must be present to minimize the risk of overlooking malignant lesions.

Thyroid nodules categorized at US examination as very low/low suspicion of malignancy according to the ATA classification system (American College of Radiology Thyroid Imaging Reporting and Data Systems [ACR TIRADS] classes 1, 2, or 3) and nodules classified as intermediate suspicion according to the ATA (ACR TIRADS class 4) with benign cytology can be considered candidates for ablation, provided cytology is benign. Two benign biopsies are usually recommended. A single benign biopsy may be sufficient for:

- nodules with very low sonographic suspicion
- autonomously functioning nodules with low to intermediate sonographic suspicion.

**FIG. 1.**
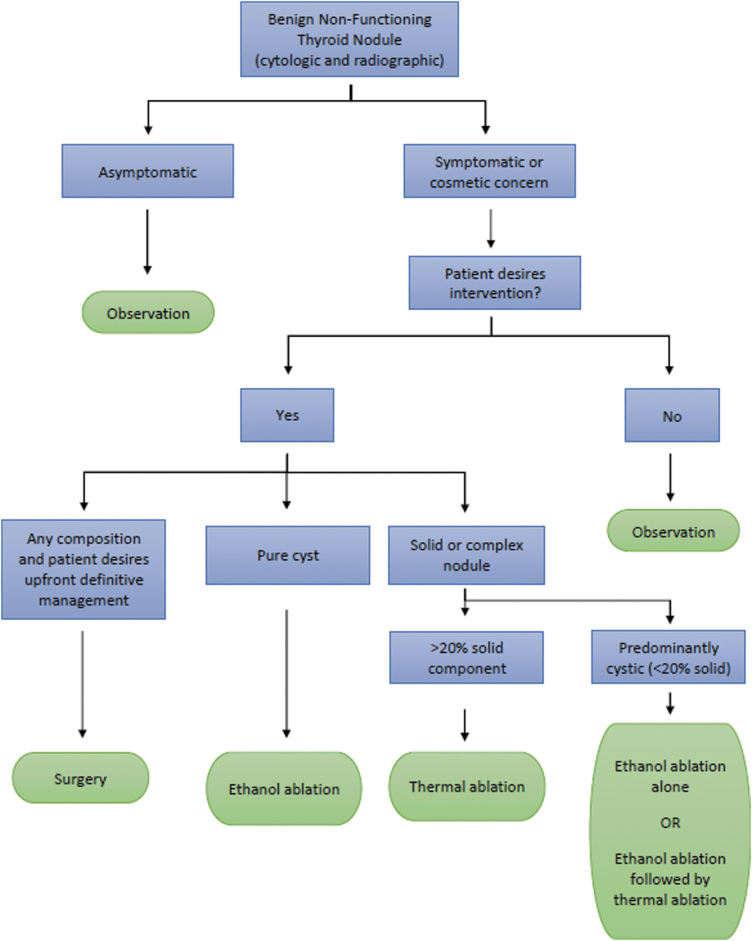
Initial management algorithm for non-functioning benign thyroid nodules.

Careful patient selection and establishment of expectations are imperative to safe and effective thyroid nodule ablations (Fig. 1). Prior guidelines have suggested nodules be at least 2–3 cm in size to consider ablation, though thresholds contributing to compressive or cosmetic concern will differ by location within the thyroid.^12,13^

Small solitary benign nodules <1.5 cm in size are rarely symptomatic and generally do not require any treatment. Though an absolute maximum size has not been established, larger nodules exceeding 20–30 mL [Nodule Volume = (Π/6) diameter 1 × diameter 2 × diameter 3] achieve less volume reduction^6,18,19^ and may require multiple ablative sessions to achieve the goals of treatment. As such, shared decision making with discussion of alternative treatment options such as surgery is essential to ensure expectations align with reasonably achievable outcomes for patients with large volume nodules.

For AFTNs, a baseline volume <10–12 mL has been suggested to optimize the likelihood of achieving euthyroidism.^20,21^ TA is only indicated in subclinical or overt hyperthyroidism caused by an AFTN. There is no evidence that ablation will cure hyperthyroidism from any other cause. A radionuclide thyroid uptake scan is generally used to determine whether a given nodule(s) is the source of excess thyroid hormone production and thus a potential target for treatment with TA.

Thyroid autoantibodies can also be useful in determining the etiology of thyroid dysfunction. A thorough evaluation of hyperthyroidism in the pre-procedural phase by a clinician who routinely manages this condition, with the commencement of anti-thyroid medications if deemed necessary, will facilitate optimal patient selection, counseling, and peri-procedural safety.

Ideally, the operator should always perform their own US examination to assess nodule characteristics and confirm procedural candidacy, as well as the absence of other lesions that may necessitate surgery rather than ablation. To maximize clinical success and safety, the entirety of the nodule should be visible on US so that it is accessible for ablation.

Although nodules extending from the inferior pole or below the sternal notch are still candidates for TA, they should be approached with caution, especially as the procedural view on US will be more subject to respiratory variation. This is especially true in patients with a larger body habitus, short neck, or difficulty with neck extension, where visualization of such a nodule is less clear.

Patients with multinodular goiters may require staged ablative procedures for adequate volume reduction of bilateral nodules and surgical intervention for these patients could offer a more timely and efficient treatment option. Patients with significant tracheal compression and/or positive Pemberton's sign on physical exam may not be best suited to ablation, as post-procedural edema or hematoma can endanger the airway.

Therefore, thyroidectomy remains the mainstay treatment for large, symptomatic multinodular goiters. However, if surgery is contraindicated or refused, a staged ablative approach or thyroid arterial embolization^22^ could be considered in carefully selected patients as adjunctive locoregional therapy for symptom palliation. RFA can interfere with a pacemaker, implanted cardiac defibrillator, or cochlear implant, and the presence of such devices is a relative contraindication to RFA, although, in such cases, bipolar electrodes or alternative TA techniques may be used.

Indications and contra-indications to TA are summarized in Table 2.

**Table 2.** Summary of Criteria for Thermal Ablation of Benign Thyroid Nodules

Minimum necessary criteria	Relative contraindications	Absolute contraindications
Dominant nodule contributing to cosmetic or compressive disturbance; OR autonomously functioning nodule causing subclinical or clinical hyperthyroidism Benign cytology on fine needle or core biopsy Ultrasound risk stratification for malignancy categorized as very low to intermediate Lack of personal risk factors for malignancy Clear comprehension and realistic expectations of the ablation procedure, expected outcomes, potential complications, and alternatives	Ultrasonographic suspicion for malignancy Cytologically indeterminate biopsy result with negative molecular markers^a^ Papillary microcarcinoma without high-risk features^b^ Multinodular goiter with significant bilateral nodularity^c^ Significant substernal extension Vocal cord paralysis contralateral to ablative side Pregnancy^d^ Pacemaker or implantable cardiac defibrillator^d^ Clotting or bleeding disorder On anti-coagulant or anti-platelet therapy and unable to cease pre-procedure	Cytologically indeterminate biopsy result with positive molecular markers^a^ Known malignancy >1.5 cm in size^b^ Treatment of areas not able to be visualized on ultrasound

^a^ Prospective trials are currently underway to evaluate the safety of using thermal ablation techniques in indeterminate nodules with benign molecular markers. At the current time, data remain limited and treatment of indeterminate nodules is not advised unless under clinical trial protocol.

^b^ Thermal ablation may be used safely and effectively in primary papillary thyroid microcarcinoma, but data in larger tumors remain limited and the treatment of such lesions is not currently advised.

^c^ In multinodular goiter, staged procedures could be considered.

^d^ RFA bipolar applicators and microwaves may be considered in these patient populations.

RFA, radiofrequency ablation.

##### Considerations specific to individual TA techniques

Although clotting disorders should be corrected and anticoagulants should be withheld before all ablation procedures, LA uses a thinner guide needle for access followed by insertion of the fiber coaxially and by virtue of this may pose less risk of peri-procedural bleeding than other techniques.

HIFU is the only technique that does not necessitate the insertion of an applicator but is reportedly a more painful procedure than other ablative techniques, can take longer to perform, and is highly sensitive to movement, frequently necessitating conscious sedation as compared with other techniques that are usually performed under local anesthesia.^10,14,23^ Efficacy of HIFU is less established than the other techniques; however, the choice of technique is generally a matter of proceduralist preference, training, and skill set.

###### Chemical ablation

Pure or predominantly cystic nodules treated with simple aspiration often recur.^21^ Therefore, patients with recurrent cystic lesions that cause compression or aesthetic complaint are candidates for EA. Nondiagnostic results are common in pure colloid cysts and are not a contraindication to ablation. Caution should be used in mostly cystic and hemorrhagic nodules. In these cases, a careful sampling of the cyst wall is recommended.^13,14^

Large nodules, those with especially viscous contents, or presence of multiple loculations, may limit the efficacy of EA, requiring multiple treatments and/or the addition of saline irrigation to wash out the viscous cyst contents before ethanol instillation.^24–26^

###### Combined chemical and TA

Though EA is recommended as a first-line treatment for benign cystic thyroid nodules, as the solid component increases (>20%), the efficacy decreases. Thus, when the nodule is of mixed composition with >20% solid component, RFA or a combination of EA and RFA may be considered.^27,28^ Aspiration and EA of the cystic portion followed by RFA, either in a single or in successive sessions, can be advantageous to minimize intranodular hemorrhage.^29^ Eligibility and contraindications are similar to those for TA.

### Patient counseling and ethics

Patient counseling is an integral part of the informed consent process before proceeding with an ablative procedure. A thorough discussion addresses benefits and risks, alternative management strategies, and sets realistic patient expectations. Alternative management options to ablation include observation, radioactive iodine for functioning nodules, and surgery, and their relative advantages and disadvantages should be presented without bias such that the patient can make an informed, individual treatment decision.

Benefits of ablative procedures include reduction in nodule volume,^1–5^ improvement in compressive symptoms and cosmetic concerns,^1–5,19^ avoidance of scars and thyroid hormone supplementation,^1–3^ improved health-related and thyroid-specific quality of life,^4,5^ and performance in an outpatient setting without the need for general anesthesia.^2^

Compared with surgery, further benefits include less recovery time and faster return to normal daily activities.^1–5,19^ However, ablative techniques also harbor novel and unique limitations compared with traditional surgical approaches or observation. Risks specific to ablation procedures should be emphasized and discussed, including thermal or chemical injury to the recurrent laryngeal nerve (RLN) and other vital structures, the need for conversion to open thyroid surgery in the case of uncontrolled peri-procedural bleeding, nodule rupture, failure to correct thyroid function for autonomous nodules with an elevated risk of hyperthyroidism relapse, delayed diagnosis of missed malignancies, regrowth of treated nodules with the possibility of future ablative procedures or traditional surgery, and the need for long-term monitoring with US.^12–14,30,31^

Patients should also be made aware that they may experience discomfort during the procedure (if not performed under deep sedation), which can often be mitigated with additional local anesthetic.

Patients on anticoagulant therapy should be advised that they will generally need to cease their anticoagulant medication before ablation, with cessation of warfarin 5 days prior, anti-platelet agents 7–10 days prior, and direct oral anticoagulants (e.g., apixaban) 24–36 hours prior.^32^

The specific anticoagulation indication will determine whether the patient requires bridging anticoagulant therapy, at the discretion of their prescribing physician, and pre-procedural laboratory tests may be necessary in some cases to check coagulation factors before ablation. Resumption of anticoagulant medication can generally occur on the day following ablation.

### Choice of location and anesthetic

A number of patient and provider factors assist in determining optimal procedure venue and anesthetic choice. A thorough medical history, with particular attention to cardiopulmonary conditions, clotting disorders, anticoagulation, and pregnancy status should be obtained and significant comorbidities or anticipated bleeding risk are indications for continuous telemetry monitoring.^33^

Patient cooperation, comorbidities, ability to interact, and procedural tolerance should be considered and, although local anesthesia with injected lidocaine provides adequate analgesia for most patients, conscious sedation administered by an anesthesiologist or other provider with the appropriate authorization and credentials may occasionally be required.

Patients who are unable to cooperate in a conscious state, tolerate a low degree of discomfort or prolonged recumbency, or suffer with significant anxiety may be better served by clinicians who perform thyroid ablation under moderate or deep sedation, or be referred for surgery.^34^ Anxiolytics, such as low-dose alprazolam or lorazepam, can be administered before the procedure per patient preference to optimize tolerance.^35^

A safety checklist of pre-procedural considerations is presented in Table 3.

**Table 3.** Ablation Safety Checklist for Early and Late Pre-Procedural Workflow

Early pre-procedural - *Patient selection* ○ Symptomatic patient	
○ Low to intermediate risk ultrasound features	
○ Benign cytology	
○ Patient desires treatment	
○ Patient has realistic expectations with regards to what one session of ablation can achieve	
○ Relative and absolute contraindications to ablation explored	
○ Patient has normal voice ± vocal fold mobility	
○ Allergies recorded in notes	
○ Does the patient have an increased bleeding risk?	
▪ If so, management plan discussed with treating physician	
- *Follow-up planned*	
○ Follow-up schedule discussed with patient	
Late pre-procedural	
- *Equipment and Technical Considerations*	
○ Active tip size chosen based on nodule size/volume	
○ Generator and pump checked and working	
○ Optimal energy to be delivered to nodule calculated based on nodule volume (optional)	
○ Starting power chosen based on active tip size and nodule characteristics	
○ Cold saline available in fridge in adequate amounts for electrode cooling (RFA)	
○ Anxiolytic medication taken by patient if required	
- *Patient and Ablation Approach Considerations*	
○ Consent signed	
○ Nodule ablation location confirmed with patient	
○ Patient re-counseled regarding normal versus abnormal expectations and symptoms during the procedure	
○ All disposable equipment available, including cold dextrose 5% for hydrodissection off critical structure and for injection in case of laryngeal nerve injury, sterile ultrasound probe covers	
○ Maximal safe volume of local anesthetic calculated by patient body weight and type of anesthetic used (most commonly 1% lidocaine)	
○ Patient positioned with neck extended	
○ Neural structures (vagus, MSG), carotid sheath, and neck viscera visualized; need for hydrodissection off nodule determined	
○ Danger zones identified	
○ Entry angle of technique applicator to skin determined based on nodule size and location in thyroid; anterior neck veins identified on ultrasound	

MSG, middle sympathetic ganglion.

### Peri-procedural

#### Equipment and technical considerations

EA as a single modality technique is effective for predominantly cystic nodules. Local anesthesia injection to skin and subcutaneous tissues is recommended to reduce pain associated with needle insertion. The needle is inserted into the center of the cyst under US guidance. A trans-isthmic approach may help prevent anterior ethanol extravasation after the needle is removed. Complete aspiration of the contents is performed with a 16–25 G needle according to the viscosity of the fluid content, keeping the needle in place and under direct visualization to avoid additional puncture of the capsule (retention of a small amount of cystic fluid can help prevent inadvertent capsule puncture).

Ethanol 95–99% is then injected into the collapsed space, observing the echogenic blush of the ethanol instillation on US. Alternatively, the cyst can be irrigated first with saline, then re-aspirated before injecting the ethanol. In addition to flushing away debris, this serves the added benefit of confirming needle placement before injecting ethanol.

Because peri-thyroidal ethanol leakage due to capsule puncture and diffusion of ethanol outside the cyst cavity may cause significant discomfort or injury to the surrounding neck structures, injection should be gradual, with a volume of 30–50% of the aspirated contents. If the patient complains of pain or “burning” during any injection of ethanol, it may be a sign of extravasation. The position of the needle and/or any evidence of extravasation should be confirmed before proceeding further. The injected ethanol should be aspirated if the symptoms get worse or not manageable with sedatives.

Radiofrequency generators for use in ablation of thyroid nodules work in conjunction with thyroid-specific electrodes.^8,36^ The generator provides continuous radiofrequency power, typically between 5 and 80 W. The electrodes currently available are monopolar or bipolar, 18- or 19-G, 7–10 cm in length, and consist of an internally cooled needle with an active tip of varying sizes (3–15 mm). Electrodes with larger active tips are typically used with a higher wattage (Table 4).

**Table 4.** Energy Settings and Choice of Radiofrequency Ablation Electrode Active Tip Size

RF power (W)	Active tip size (cm)
RF power according to active tip size	
5–15	0.38
10–30	0.5
20–40	0.7
30–80	1.0
50–90	1.5

RF, radiofrequency.

The wattage chosen for ablation of an individual nodule varies based on many factors such as the size of the nodule, the size of the active tip, the specific region being treated (lower near critical structures), patient tolerance, and operator comfort. When using monopolar electrodes, grounding pads must be placed on the skin to complete the circuit and safely return electric current from the patient back to the generator through a cable.

Monopolar electrodes should not be used in pregnant women or patients with implanted electrical devices. A peristaltic pump is used to circulate chilled saline (close to 0°C) through the electrode; this continuously reduces the temperature around the electrode to prevent or minimize carbonization around the electrode tip. RFA of thyroid nodules is performed under real-time US guidance and requires a linear transducer with a frequency range between 7 and 14 MHz.

LA is achieved through an interface (Electrospray Ionization) between the laser source and the US machine, and a bracket is mounted on the probe. This houses a multi-pierced guide for the insertion of guide needles with fibers, with three different angles to be chosen at 65°, 70°, and 75°. Once the planning has been performed and the final energy to be delivered has been chosen (from 1200 to 1800 J per illumination and per fiber depending on the volume to be treated), the fiber(s) is/are introduced into the 21-G spinal guide needles under US guidance with a direction along the longitudinal axis of the neck.

The laser light generator is set to 3 W, with gradual initial increases up to 5 W to be modulated depending on the tissue response. For each milliliter of tissue to be treated, the energy should be at least 500–600 J/mL with a maximum of 800 J.^7,8^ Each single laser illumination should not exceed 1800 J of total delivered energy. Laser safety precautions should be utilized at all times by all team members. Once inserted, laser fibers can be retracted one or more times to cover the entire volume of the nodule (the “pull-back technique”), which reduces the number of direct punctures of the nodule.

The MWA systems consist of a microwave generator and a 16–18 G internally cooled needle/antenna. The active portion of the microwave antenna can vary between 3 and 5 mm, and the shaft is typically 10 cm in length. The MWA creates heat via the production of an electromagnetic field. Compared with other TA techniques, the treatment zone is ablated in a more homogenous pattern, with reduced production of micro-bubbles and thus reduced effacement of the treated area on US.

The moving shot technique can be applied with a single antenna and very low power can be delivered (10–20 W) with thinner antennas. Ablation is sequentially performed in 5–10 second periods to treat the entirety of the nodule and continues until the nodule demonstrates appropriate hyperechoic echotexture changes. Optimal assessment of the limits of the ablated zone requires specific competency in the MWA technique.

Previous studies have reported relatively short total ablation times ranging from 32 to 180 seconds. For large volume nodules, a 16 G antenna can be used with a “steady technique” in multiple steps: the needle is inserted into the area to be treated and appropriate power applied (20–40 W) according to an algorithm available with each generator.

Once a predictable necrotic area of up to 3 cm dimension is produced, the needle is retracted and positioned in a new area until the entire nodule has been treated. This technique avoids inserting multiple antennas into the nodule, a process that has been associated with increased complications.

The HIFU machine is a US-guided device that has an energy generator, a treatment head that comprises an imaging transducer (7.5 MHz, 128 elements, linear array), and a treatment transducer (3 MHz, single element, 60 mm in diameter).^37,38^ Once the treatment head is placed over the side of the target nodule, the imaging transducer allows the guidance of the treatment beam to the center of the target nodule.

Safety of energy delivery is facilitated by the computer-driven nature of this treatment, which utilizes a laser beam to detect neck movement and immediately stop US wave delivery. The ablation begins at 280 J/pulse and the power is gradually increased until hyperechoic marks appear at the focal point, then that level of energy is maintained throughout the entire treatment cycle. The treatment cycle is semi-automated with the treatment head moving slowly across the entire nodule until it is ablated in its entirety.

### Techniques to optimize peri-procedural safety

#### Anesthesia

During the procedure, optimizing the safety of proximate anatomic structures is paramount. With local anesthesia, the patient is fully awake and able to vocalize to ensure intact laryngeal nerve function and provide feedback regarding discomfort, which is immediately relayed to and can be acted on by the operator. Conscious sedation may minimize patient anxiety; however, timely communication and recognition of concerns or side effects can be less reliable depending on the depth of sedation.^39^

The most common approach for local anesthesia injection utilizes a small gauge (27–30 G) needle to inject the anesthesia subcutaneously in the midline neck. Subsequently, a wider bore needle (25–27 G) connected to a syringe containing additional local anesthetic is inserted through this anesthetized area, directed to the thyroid capsule, and advanced between capsule and muscle to bathe the capsule in anesthetic.

Anesthesia volume is determined by nodule size and patient weight. Lidocaine 1% is a commonly used anesthetic. Doppler interrogation of superficial neck structures must be done before needle insertion to identify and avoid injury to the anterior jugular veins and thyroid vessels. If a vessel is punctured, a hematoma may result on needle withdrawal. Firm neck pressure over the puncture site usually controls any bleeding and allows for safe continuation of the ablation procedure.

#### Trans-isthmic approach and moving shot technique

The trans-isthmic approach offers distinct safety advantages for operators treating patients with RFA and MWA. First, the operator can monitor the association between the applicator, target nodule, and RLN, which is situated between the trachea and thyroid gland. In the upper neck, near the cricothyroid joint, the RLN can be protected by applying the concept of a “danger triangle” as previously described.^6,8,31,36^

**FIG. 2.**
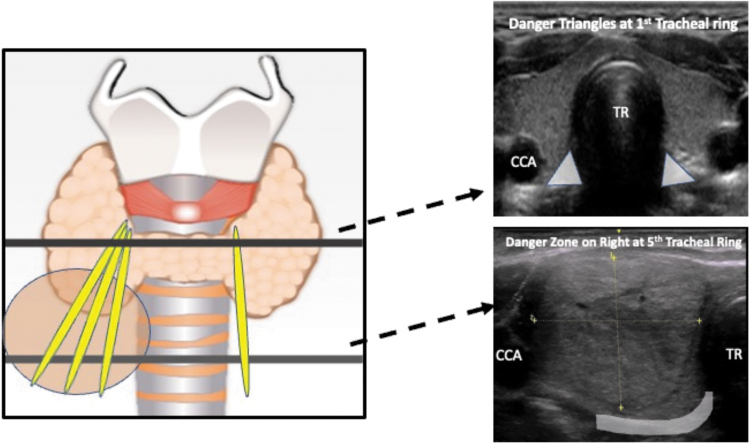
The danger triangle concept is applicable to ablation at the level of the upper tracheal rings and cricothyroid joint. In the lower neck, the concept of a “danger zone” is more appropriate as the recurrent laryngeal nerve is often laterally placed at these levels, especially on the right. CCA, common carotid artery; TR, trachea.

At the level of the mid-cervical trachea and below, the concept of the “danger triangle” for RLN safety becomes less reliable, particularly on the right side where the nerve can run 1–2 cm lateral to the tracheoesophageal groove.^40^ At these lower cervical levels, the entire posterior capsule should be considered a “danger zone” and precautions taken to minimize heat transmission to this region (Fig. 2).

Second, the normal isthmic parenchyma between the target nodule and the applicator (i.e., electrode with RFA, antenna with MWA) insertion site prevents the leakage of hot ablated fluid to the perithyroidal area, which could otherwise cause pain and tissue damage. Finally, the applicator position remains stable even when a patient talks or coughs, thereby minimizing the risk of injury to surrounding structures.^36^

The moving-shot technique divides the nodule into multiple small ablation units.^6^ These ablation units can be treated individually and systematically. To prevent limitation of the acoustic window by gas bubbles, the applicator tip should be initially positioned in the deepest and most remote portion of the nodule, with subsequent retraction to more superficial areas. The location of the applicator tip should be continuously monitored via real time US during the procedure to prevent possible thermal damage to adjacent critical structures.

#### Hydrodissection

When the target nodule abuts critical structures, hydrodissection using 5% dextrose solution in water (D5W) should be considered.^6,8,30,36^ Slow injection of D5W creates a barrier between the target nodule and critical structures. This may need to be repeated multiple times depending on the length of the ablation. Given that thyroid tissue deep to the capsule is nearly devoid of sensory innervation, the sensation of pain on the part of the patient could be an indicator of potential trauma to critical structures.

The application of local anesthesia adjacent to critical structures such as the trachea should, therefore, be avoided so that the pain reflex can improve safety. For the same reason, avoiding general anesthesia or conscious sedation may enhance the safety profile and should be encouraged for operators with limited experience.

#### US-guided techniques for thyroid vascular ablation

Artery-first ablation is effective to minimize the heat-sink effect in hypervascular nodules. The concept is similar to that of thyroid arterial embolization, in that direct puncture and destruction of the feeding artery by ablation is performed before ablation of the nodule parenchyma, allowing for lower currents to be utilized during parenchymal ablation and thus reducing the risk of heat transmission to surrounding critical structures.

The counterpart, marginal venous ablation, is a technique to destroy veins around the thyroid nodule. Destruction of a marginal vein may help achieve rapid volume reduction at the early follow-up period by maximizing the initial ablation zone as well as minimizing marginal regrowth at long-term follow-up.^41^ When used together, arterial ablation should precede venous ablation.

#### Management of peri-procedural complications

Power used adjacent to critical structures should be carefully monitored, with lower power utilized if ablating near neck viscera, in the posterior danger zone or superiorly near the external branch of the superior laryngeal nerve.^40^ If vocal hoarseness or sudden cough is noted during the procedure and there is concern for RLN injury, tracheal injury, or esophageal injury, ablation should be halted.

A bolus of cold D5W can be injected into the perithyroidal region adjacent to the ablated area where the hoarseness occurred, as it can potentially dissipate the heat already transmitted to the nerve.^30,36^ If the decision is made to continue with the ablation, the ablated area where the voice change occurred should be avoided for the remainder of the procedure. Hemorrhage is usually self-limited and controlled with simple compression of the hemorrhagic portion.

In the case of large hemorrhage directly from an artery, direct ablation of the injured artery is the treatment of choice if the artery can be detected and is of small enough caliber to ablate. For more profuse bleeding, endovascular or surgical intervention is needed. Lidocaine toxicity is a rare complication. Early signs include perioral numbness, tinnitus, and agitation. With larger doses, muscle twitches and seizures are possible.^42^

To minimize lidocaine toxicity, it is recommended to use <4.5 mg/kg of 1% lidocaine. Treatment is conservative in mild toxicity cases, with consideration for lipid emulsion therapy with lipofundin 20% and transfer to a higher level of care as appropriate.^43^

### Post-procedural

#### Criteria for safe discharge

Complication rates following thyroid nodule ablation are low as reported in international series and corroborated by the small number of North American series reported to date.^12–14,21.29,30,44–46^ To detect an early complication, all patients should be observed for a period of at least 30 minutes following ablation, or longer if sedative medications are utilized.

Vital signs, including heart rate, blood pressure, and oxygen saturation, should be monitored. The patient's breathing should be assessed for stridor, cough, or respiratory distress. The patient's voice should be assessed to confirm it is comparable with pre-procedure, as recurrent laryngeal or vagus nerve injury can result in vocal hoarseness and decreased ability to project the voice above background noise.

If there is concern for voice change, US or laryngoscopy assessment of the vocal cords should be performed, with laryngoscopy being the gold standard examination modality. Laryngeal nerve injury can also cause patients to cough when swallowing liquids and, if nerve injury is suspected, patients should be given a sip of water to assess whether they are able to swallow normally and to gauge their risk of laryngeal aspiration.

Patients should be counseled on identifying early complications such as expanding hematoma, and when to seek urgent medical care. Patients are instructed to avoid submerging the access site or sites in water for 24 hours. Patients are encouraged to avoid strenuous physical activity for 2–3 days following the procedure, and to avoid pressure or trauma to the neck. The neck puncture site should be checked before discharge to assess for hematoma or bruising. If conscious sedation or anxiolytic medications were used, the patient will require assistance with transportation home.

In the absence of conscious sedation, patients should still be encouraged to arrange for a ride home due to potential soreness after the procedure. Patients who require air transport should be advised to stay in a local hotel for the first 24 hours following the procedure. Routine use of antibiotics is not required unless perioperative complications are detected.

#### Immediate safety considerations (up to72 hours)

After thyroid nodule ablation, pain and soreness in the neck are expected. In addition, some swelling of the nodule and surrounding tissues is common during the first week. These symptoms will usually peak in the first 3–5 days following the procedure. In most cases, pain and inflammation can be managed with ice packs and over-the-counter analgesics (e.g., ibuprofen and acetaminophen).^13^

Patients rarely require opioid pain medications and their use should be avoided. A steroid taper may alleviate discomfort in the case of pain or early compressional symptoms due to initial nodule volume increase.

Early complications are usually a result of injury to critical structures that surround the thyroid gland, such as the esophagus, trachea, RLN, carotid artery, vagus nerve, and cervical sympathetic ganglion.^19,47,48^ Since heat, and therefore injury, can extend for a distance of 1–5 mm beyond the applicator tip (depending on the active tip size and the power setting), advancement of the tip must take into account proximity to vital structures.^27^

Injury to the trachea or esophagus may result in subcutaneous emphysema and neck infection. Appropriate recognition, with imaging studies and immediate management based on extent of injury, is crucial.

As with surgery, the proximity of the RLN to the thyroid gland and central compartment lymph nodes places it at risk for injury during ablation procedures. Studies examining rates of RLN injury utilizing pre- and post-procedural laryngoscopy are lacking, thus accurate rates of RLN injury are difficult to ascertain. A systematic review and meta-analysis identified a 1.44% overall rate of transient or permanent voice change following RFA.

The rate of voice change was higher (7.95%) in the subset of 176 patients undergoing RFA for recurrent thyroid cancer, primarily in the central compartment.^47^ Injuries to other nerves such as the vagus, sympathetic chain, brachial plexus in the supraclavicular fossa, and phrenic nerve have been documented but are rare and such injury may be prevented by careful pre-procedural evaluation of the vagus nerve and middle sympathetic ganglion location.^19,47,48^ If peri-procedural injury is suspected, evaluation of these structures should be performed immediately as detailed in the Discharge section earlier.

#### Short-term safety considerations (3 days to 1 month)

Compared with surgical management, it is important to educate patients that the benefits of ablation are not immediate, and instead accrue over the course of months. The nodule size reduction post-ablation within the first month may be limited. Despite this, patients often report significant symptom improvement in the first month.

Meta-analysis of outcomes in RFA for benign thyroid nodules reported a pooled volume reduction of 64.5% at 6 months and 76.9% at 12 months, followed by further volume reduction of 92.2% at 36 months.^48^ This was accompanied by improvement in nodule-related symptoms and cosmesis.^49^ Volume reductions for the various forms of TA in major trials to date are summarized in Appendix Table A1.

Transient hyperthyroidism following nodule ablation can occur, especially following ablation of an AFTN. Patients may complain of symptoms including heart palpitations, anxiety, insomnia, and myalgia. Hyperthyroid symptoms generally settle 2–4 weeks post-ablation and rarely necessitate the commencement of antithyroid medications.^13,50^

However, as the thyrotoxic state is mainly due to a destructive thyroiditis, the use of beta-blocking agents may effectively control mild thyrotoxic symptoms. If patients are already on antithyroid medications pre-procedure, it is recommended that they continue these in the early post-procedural period for at least 3–4 weeks or until blood tests have normalized.

Transient thyroiditis can also occur following ablation.^3^ Patients may experience low grade fevers, malaise, aches, or worsening compressive symptoms. After excluding a hematoma with US or cross-sectional imaging, a steroid taper can be considered for these patients.^44^

Nodule rupture is an uncommon but significant risk of TA thought to be caused by either delayed bleeding or liquifactive necrosis extruding into the peri-thyroidal region, predominantly anteriorly. Patients most commonly present with sudden neck swelling and pain at 2 weeks to 3 months post-ablation.^51^ Peri-procedural risk factors for rupture remain unclear at this time due to the small number of patients affected worldwide and thus the focus must be on identification and management, rather than prevention.

Diagnosis can generally be made with US alone. In the absence of airway obstruction, initial management is observation. Antibiotic treatment should be considered, and a steroid taper can help to decrease pain and swelling. If such conservative management is not successful in decreasing the size of the mass and relieving symptoms, aspiration, incision and drainage, or rarely surgical resection may be required.

#### Long-term safety considerations (>1 month)

In addition to nodule rupture, thyroid function changes are rare late complications of ablation. While transient thyroid function test abnormalities can be detected in the first few weeks (see Short term safety considerations above), clinically significant hypothyroidism typically manifests between 1 and 12 months.^28,52–54^ As such, all patients who develop early hypo- or hyperthyroid symptoms after ablation should undergo repeat thyroid function testing at >1 month post-procedure.

Nodule regrowth and need for more than one ablation are long-term considerations that patients must be counseled about before undergoing ablation. Although regrowth definitions in the literature vary, the risk of regrowth after TA is 5–40% and increases the larger the baseline nodule volume.^30,55–58^

Regrowth most commonly occurs in undertreated peripheral areas where the proximity of critical structures (RLN, carotid sheath, vagus nerve, sympathetic ganglions) limits power usage. Generous hydrodissection in these areas can displace critical structures off the thyroid capsule and facilitate ablation efforts, and vascular ablation techniques can reduce hemorrhage risk and enable lower power usage near critical structures.

#### Follow-up safety considerations

Long-term follow-up with clinical, laboratory, and sonographic evaluation is recommended following the ablation of thyroid nodules; however, the frequency and duration of monitoring reported in existing literature varies.

##### Biochemical evaluation

Overall, hypothyroidism following chemical or TA of thyroid nodules is rare and is thought to develop due to the progression of autoimmune thyroiditis associated with pre-existing thyroid antibodies. Therefore, prolonged, serial biochemical evaluation of thyroid function is not recommended post-procedure except for hyperfunctioning thyroid nodules.

Following TA of hyperfunctioning thyroid nodules, thyrotropin (TSH), free thyroxine, and triiodothyronine should be measured at each follow-up to assess whether medical therapy (antithyroid medications and/or beta-blockers) can be reduced or discontinued.

##### Sonographic evaluation

Follow-up neck US is typically recommended at 1–3, 6, and 12 months post-ablation to assess ablation outcomes, including volume reduction, nodule appearance, nodule vascularity, and areas at risk for regrowth. Volume reduction ratio (VRR) is calculated [(Baseline Volume − Final Volume)/Baseline Volume × 100]. It is important that the operator is familiar with post-ablative sonographic nodule changes, such as hypoechogenicity, border irregularities, and the presence of hyperechoic foci following RFA, to avoid unnecessary intervention.

Information handouts describing post-ablation nodule appearance should be provided to patients and external physicians/care providers to avert unnecessary worry and/or interventions. As maximal nodule volume reduction is typically achieved in the first year following TA, subsequent sonographic examinations can be undertaken annually to monitor for nodule regrowth that can occur 3–5 years following the procedure.

The neck US can be performed at bedside during a clinic visit or by a radiologist. Failure to achieve expected volume reductions at any stage during the follow-up period should prompt re-evaluation by the treating physician or multidisciplinary team with regards to appropriate subsequent management planning.

##### Considerations for additional ablative sessions

With a single treatment of TA, optimal volume reduction is generally obtained for smaller nodules with baseline volumes <10–20 mL. For large nodules >20–30 mL in volume, nodules failing to demonstrate adequate reduction in volume (e.g., more than 30% by 6 months following the procedure), nodules demonstrating regrowth in previously untreated peripheral areas and/or persistent or new patient compressive symptoms, consideration should be given to repeat ablative session(s) versus referral for surgical intervention.

In such cases, fine needle aspiration (FNA) of the undertreated portions or of areas of marginal regrowth can be considered, although pathologic interpretation can be challenging. Patient clinical factors, such as comorbidities, as well as preferences, such as desire to avoid hypothyroidism, should be taken into account when deciding next steps in management.

Emerging evidence suggests that residual unablated volume may be a predictive factor for regrowth that could guide decision making regarding the necessity of re-treatment.^54^ Studies comparing RFA to laser TA suggest that risk of regrowth and additional treatments is lower with RFA.^8,59^ For large bilateral thyroid nodules selected for TA, a staged procedure is recommended to avoid possible airway compromise due to post-procedure swelling that is directly correlated to the size of the ablation area.

Generally, the largest nodule (or nodules) on one side is treated in the initial ablation session followed by staged contralateral nodule ablation, with timing of the staged procedure determined by discussion between the proceduralist and the patient. Vocal fold motion should be checked to ensure bilateral mobility before second or subsequent ablation procedures.

### Ablation techniques: prior skillset, knowledge, and training for safe performance

#### Learning curve data

There is a learning curve to performing ablation that clinicians need to master before they can provide optimal treatment to their patients with low morbidity and high efficacy. To the best of our knowledge, three reports to date have focused on learning curves for thyroid RFA^60–63^ and one study addressed more specifically the issue of complications.^63^

Two of the three studies focused on the learning curve for a single operator, and the other involved a team of two radiologists. Of note, all interventionalists were highly experienced in head and neck US and FNA biopsy before performing RFA and thus the learning curve may have been shorter than for less experienced proceduralists. The overall rate of complications during the learning phase was low (1.6%).

Of the 291 patients included in the 3 studies, 3 transient vocal cord palsies (1.0%), 1 nodular rupture (0.3%), and 1 vasovagal reaction terminating the procedure (0.3%) were recorded. In a multicenter study by Baek et al., operators were divided in two groups comprising less-experienced physicians who had performed <50 ablations versus experienced operators who had performed >100 ablations.^30^

The major complication rate (voice change, nodular rupture, brachial plexus injury) was significantly lower in patients treated by experienced operators than in patients treated by less-experienced operators (0.7% vs. 2.9%, *p* = 0.007). The total complication rate was also lower for experienced operators than for less-experienced operators, but this difference was not significant (2.0% vs. 3.9%, *p* = 0.051).

All publications showed that clinical efficacy was obtained after treating ∼30 cases (initial learning phase). There was continued improvement in standard outcome parameters (technical efficacy, VRR, and initial ablation ratio) until ∼60 RFA procedures had been performed (consolidation phase). Further optimization of the ablation ratio, durability of volume reduction, and prevention of regrowth occurred after more than 60 procedures (proficiency phase).

In the proficiency phase, proceduralists gained expertise in increasing the energy delivered per mL of tissue ablated, treating the marginal part of the nodule,^53^ and balancing this marginal nodule treatment with the associated increased risks of thermal injury to adjacent structures.

It must be noted that, in addition to proceduralist skill, final volume reduction achieved was also dependent on disease factors such as baseline nodule volume, nodule composition, and vascularity.^18^ As such, we recommend that clinicians select moderate size (<20–30 mL), non-vascular nodules with favorable characteristics and location, as their initial ablative cases.

#### Prior knowledge and qualifications

To be qualified to perform ablation of benign thyroid nodules, a practitioner should be board certified or eligible in an appropriate medical specialty (Otolaryngology Head and Neck Surgery, Interventional Radiology, General Surgery with a focused practice in Endocrine Surgery, Endocrinology) and have extensive background knowledge and clinical experience in (1) the clinical diagnosis and treatment of thyroid nodules; (2) neck imaging anatomy; (3) thyroid US imaging and FNA biopsy procedures; and (4) US risk stratification for benign and malignant thyroid tumors.

The subsequent preclinical training should include extensive ablation of phantom models. Thereafter, initial thyroid nodule ablations on patients should be performed under the guidance of a proctor experienced in thyroid TA. In addition, it may be helpful to organize a presentation on the technical capabilities of the selected equipment from the manufacturer, covering the logistics of the generator system and troubleshooting algorithms for equipment issues.

The number of procedures done by the applicant to achieve competency should be determined by the appropriate department chair or division chief and, if local health authorities require specific qualifications to perform ablation treatment, these qualifications should be obtained before initiating an ablation program.

In accordance with the learning curve data presented earlier, physicians with existing expertise in US and FNA will pass the initial learning phase independently after performing at least 20–30 cases of thyroid ablation. The impact of phantom practice and proctoring on the individual achievement of competency remains to be objectively studied; however, both practices are highly recommended as noted earlier. Participation in education and basic training courses offered by professional thyroid societies and associations on the various TA techniques is also recommended.

To achieve proficiency, physicians should participate in patient management from pre-procedural diagnosis, evaluation of treatment indications and methods and discussion of possible risks and countermeasures, to perioperative treatment, treatment of postoperative complications, and follow-up. A detailed record of procedural specifics, complications, and outcomes should be maintained by all practitioners performing ablation for self-audit purposes.

### Ablation techniques: strategies to optimize safety

#### Multi-disciplinary programs

Patients treated with ablative therapies are often medically complex and therefore require both comprehensive pre-treatment evaluation and longitudinal post-treatment follow-up. Since all aspects of needed care may not be provided by the proceduralist offering ablative therapies, a multidisciplinary approach to care is often optimal and should involve medical, surgical, and radiologic specialties as needed.

It is, therefore, incumbent on providers to understand any limitations of the care they offer and develop collaborations with colleagues from additional specialties as needed to ensure all aspects of pre-treatment evaluation and post-treatment follow-up and interventions, if necessary, are provided.

Examples of scenarios where a multidisciplinary approach to care can be of particular benefit include:

- Large retrosternal nodules with visceral compression where surgical excision may be preferred over ablation
- Evaluation and management of pre- and post-ablation thyroid function
- Regrowth of a previously ablated nodule where retreatment versus surgical excision or an alternate mode of treatment may represent best possible care
- Management of new nodules post-ablation
- Post-treatment follow-up so that the unique post-ablation ultrasonographic changes typical of treated lesions may be followed and interpreted correctly
- Consideration for treatment of primary or recurrent thyroid carcinoma in patients who are not good surgical candidates.

#### Health care setting and administration themes

In his popular “Diffusion of Innovations” theory first published in 1962, famous sociologist and communications theorist Everett Rodgers proposed that the successful adoption of an innovation is dependent on the infrastructure of the social system in which the adoption process occurs.^64^ A critical component of safely and successfully implementing a TA program is to ensure that the procedures will be performed in a safe environment and that all aspects of the proposed operational flow have been thoroughly recognized, analyzed, and addressed.

**FIG. 3.**
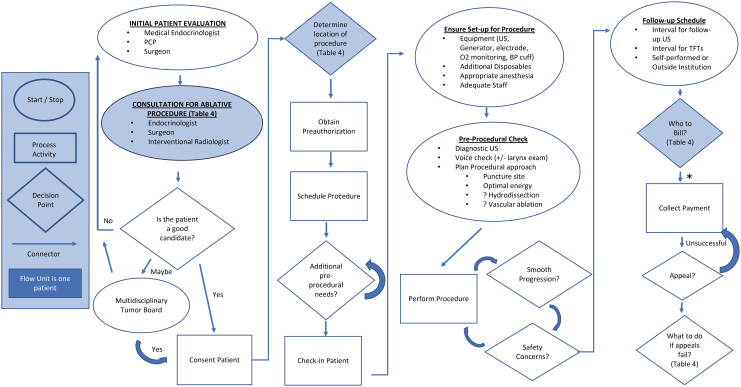
Operations and process flow chart for implementing a new TA program. A sample flow chart of the operations and processes involved in implementing a new TA clinical program is shown. To ensure safe implementation, the various points in the flow chart should all be addressed. Additional considerations of highlighted points are summarized in Table 5. *The process of billing, payment, and appeal is also a pre-procedural consideration and may occur before performance of the ablation procedure. BP, blood pressure; PCP, primary care physician; TA, thermal ablation; TFT, thyroid function tests; US, ultrasound.

Figure 3 is a sample flow chart of the operations and processes of implementing a new TA program. Table 5 contains detailed considerations for the highlighted points in the flowchart. For each step in the flowchart, exact details will vary depending on individual practice patterns and institutional resources; however, each step poses a challenge that must be satisfactorily addressed within the social system of each practice and institution in order for the new program to succeed.

**Table 5.** Organizational Considerations When Implementing a New Thermal Ablation Program

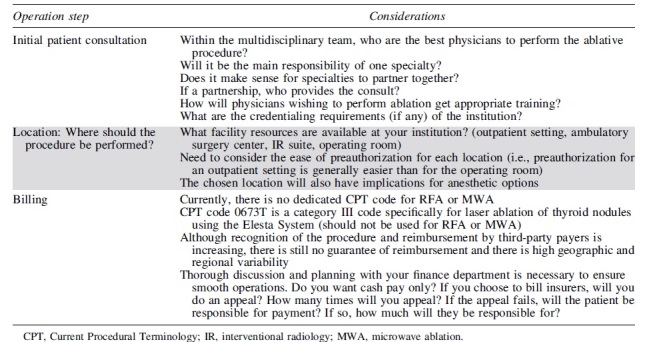

One additional important consideration that warrants mention is how to best minimize financial toxicity for the patient. Given the lack of a Current Procedural Terminology (CPT) code and inconsistency in reimbursement, charges for TA procedures are largely dependent on institutional policies and are highly variable. Two recent studies have established that RFA of benign nodules can be a cost-effective treatment if the cost of the RFA electrode is kept below $2100^65^ and the overall cost of treatment kept below $17,950 USD.^66^ Additional billing considerations for TA procedures are listed in Table 5.

## SAFETY RECOMMENDATIONS

**FIG. 4.**
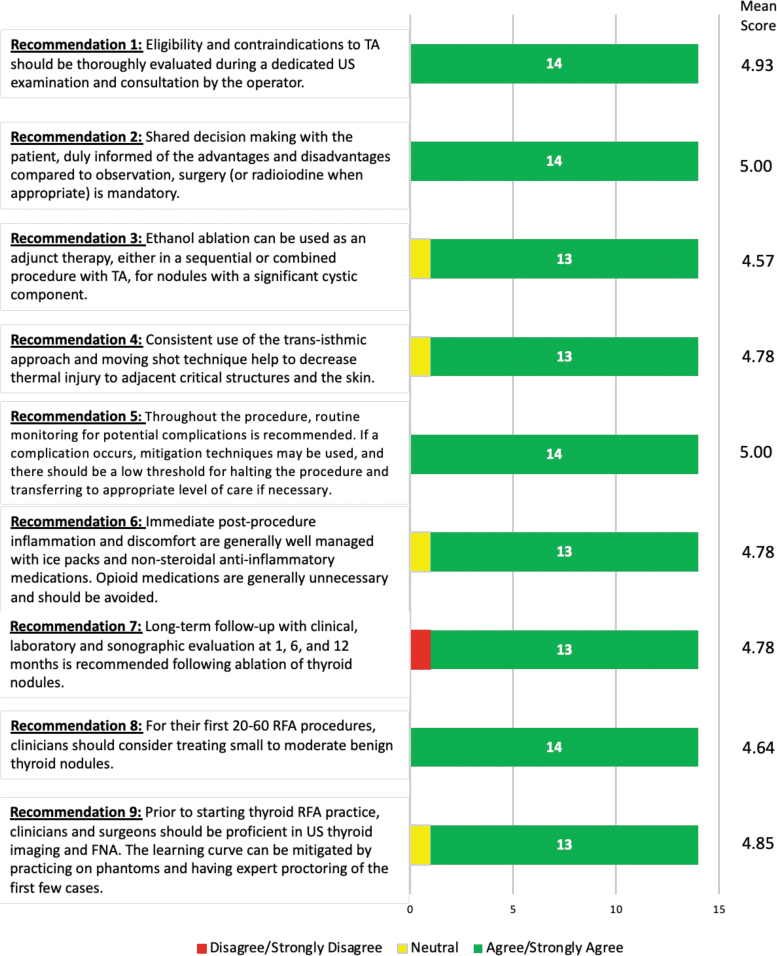
Strength of consensus from Delphi survey. FNA, fine needle aspiration; RFA, radiofrequency ablation.

In the first round of the Delphi survey, all recommendations achieved consensus except for recommendations 6, 7, and 8 (Fig. 4). These safety recommendations were revised while incorporating the feedback provided by respondents. In the second round of the Delphi survey, consensus was achieved for all recommendations (Fig. 3).

## SUMMARY

As the adoption of ablation techniques by practitioners in North America continues to increase, careful delineation of safety measures to mitigate risks is paramount. The current article provides guidelines and suggestions regarding safety considerations, risk mitigation, and prior learning for practitioners performing or intending to perform chemical or TA of benign thyroid nodules.

## Supplementary Material

Supplemental data

## Appendix

**Appendix Table A1.** Outcomes of Chemical and Thermal Ablative Therapies

First Author, Year, Country	Study Design	*n*	Vol. (mL)	VR (%)	Follow-up (months)	Comp^*^ (%)
**RFA**						
Kandil, 2022, USA^67^	RC	162	4.17	54–76	1–12	2.5
Hussain, 2021, USA^2^	RC	47	8	70.8	3.5	4.2
Kuo, 2021, USA^68^	RC	24	8.52	50.7	1.1	5
Hamidi, 2018, USA^69^	RC	14	24.2	44.6	8.6	NR
Lee, 2023, South Korea^70^	RC	287	6.4	91.9	56.5	5.2
Lin, 2022, Taiwan^71^	RC	826	21.51	72.4	6	4.8
Li, 2021, China^72^	RC	1289	7.29	77.8	12	NR
Vuong, 2019, Vietnam^73^	RC	251	6.18	81	12	0.8
Deandrea, 2019, Italy^18^	RC	215	20.9	66.9	60	2.8
Lee, 2019, South Korea^74^	RC	1619	10.2 (n, 374) and 12.85 (n, 626)	84	10	1.3
Jung, 2018, South Korea^75^	PC	276	14.2	95.3	60	4.3
Dobnig, 2018, Austria^76^	PC	222	13.8	80	12	NR
Jeong, 2008, South Korea^49^	RC	236	6.13	84.1	1–41	NR
**LA**						
Gambelunghe, 2022, Italy^77^	RC	24	138	80	48	20.8 (pain, fever)
Gambelunghe, 2021, Italy^78^	RC	171	16.7	59	120	8.2
Squarcia, 2021, Spain^79^	RC	30	18.9	60	60	9.8
Dossing, 2019, Denmark^80^	RC	110	9	85	45	NR
Gambelunghe, 2018, Italy^81^	RC	82	12	58	36	13.4 (pain)
Achille, 2016, Italy^82^	RC	45	24.2	84	12	2.2
Negro, 2016, Italy^83^	RC	56	15.7	55.5	48	7.1
Pacella, 2015, Italy^44^	RC	1534	27	72	12	0.9
Dossing, 2011, Denmark^84^	RC	78	8.2	51	38	33 (pain)
Valcavi, 2010, Italy^85^	RC	122	23.1	47.8	36	4.9
**MWA**						
Yildirim, 2022, Turkey^86^	RC	40	19	79	12	2.5
Du, 2022, China^45^	RC	148	15.6	96.9	48	3.38
Mo, 2022, China^87^	RC	53	11.68	29–92	1–18	37.7 (pain)
Zhao, 2021, China^88^	RC	53	7.28	84.64	36	3.8
Liu, 2017, China^89^	RC	474	13.07	94	12	3.8
Wu, 2017, China^90^	RC	121	8.56	85.97	12	4.1
Heck, 2015, Germany^91^	RC	34	18.15	53.6	3–6	11.7
Yue, 2013, China^92^	RC	477	2.13	38	24	4.2
**HIFU**						
Vorlander, 2022, Germany^93^	RC	75	4.4	60.7	12	3.8
Fischer, 2022, Germany^94^	RC	58	NR	63.9	12	5.2
Lang, 2019, China^95^	RC	108	10.1 (1 T) and 20.34 (2 T)	70.41	24	3.7
Trimboli, 2018, Switzerland^96^	RC	26	2.81	48	12	0
Sennert, 2018, Germany^97^	RC	19	2.56	58	3	NR
Kovatcheva, 2015, Bulgaria^98^	PC	20	4.96	48.7	6	5
**EA**						
Abdelgawad, 2022, USA^99^	RC	34	28.6	2.32	6	4.55
Steinl, 2022, USA^100^	RC	17	21.3	66–78	1–12	0
Sharma, 2020, USA^101^	RC	18	5.7	56.1	3	NR
Iniguez-Ariza, 2018, USA^102^	RC	20	19.6	75.64	24	20
Karatay, 2021, Turkey^103^	RC	46	NR	89.7	6	NR
Cho, 2021, South Korea^104^	RC	61	21.9	80.7	10.5	8.2
Hey, 2021, UK^105^	PC	26	22.3	96	14	38 (pain)
Basu, 2014, India^106^	RC	65	8.87 (n, 47) and 7.21 (n, 13)	78.3	12.3	0
Valcavi, 2004, Italy^107^	RCT	143	19	85.6	12	NR
Bennedbaek, 2003, Denmark^24^	RCT	33	8	83–100	6	21 (pain)
Verde, 1994, Italy^108^	RCT/PC	10/32	14.5	73.5/82.7	1/12	0/9.3 (pain)
**Comparisons**						
Nguyen, 2023, Vietnam^109^	RC	22 (RFA) vs. 17 (EA)	6.55 vs. 5.21	72.4 vs. 53.1	1	NR
Zhang, 2022, China^110^	RC	31 (MWA) vs. 30 (EA)	8.33 vs. 4.92	87.1 vs. 86.7	12	19.35 vs. 26.7 (pain)
Karatay, 2022, Turkey^111^	RC	46 (RFA) vs. 50 (EA)	21.41 vs. 20.52	80.8 vs. 70.7	6	6.5 vs. 8
Bernardi, 2020, Italy^112^	RC	216 (RFA) vs. 190 (LA)	14.3 vs. 12.2	77 vs. 57	60	NR
Cesareo, 2020, Italy^59^	RCT	30 (RFA) vs. 30 (LA)	26 vs. 24.7	63.3 vs. 53.2	6	16.7 vs. 16.7
Pacella, 2017, Italy^113^	RC	152 (RFA) vs. 449 (LA)	49 vs. 45.1	59.6 vs. 62.8	12	14.5 vs. 9.6
Cheng, 2017, China^114^	PC	687 (RFA) vs. 664 (MWA)	7.22 vs. 7.72	89.6 vs. 81.1	13.5 vs. 13.9	4.78 vs. 6.63
Yue, 2017, China^115^	RC	158 (MWA) vs. 102 (RFA)	4.6 vs. 5.7	77.2 vs. 79.4	12	5.9 vs. 3.9
Baek, 2015, South Korea^116^ (cystic nodules)	RCT	22 (RFA) vs. 24 (EA)	8.6 vs. 14.7	87.5 vs. 82.4	6	0 vs. 4
Sung, 2013, South Korea^117^ (cystic nodules)	RCT	21 (RFA) vs. 21 (EA)	9.3 vs. 12.2	93.3 vs. 96.9	6	0 vs. 0

^*^ Complications—major and minor—as defined by the Society of Interventional Radiology criteria or as reported in publication.

EA, ethanol ablation; HIFU, high-intensity focused ultrasound; LA, laser ablation; m months; MWA, microwave ablation; n, number of nodules; NR, not reported; PC, prospective cohort; RC, retrospective cohort; RCT, randomized controlled trial; RFA, radiofrequency ablation; T, treatment session; Vol, Basesline nodule volume; VR, volume reduction; UK, United Kingdom; USA, United States of America.
