# Supplementary material for: General Principles for the Safe Performance, Training, and Adoption of Ablation Techniques for Benign Thyroid Nodules: An American Thyroid Association Statement
Source: Thyroid. 2023 Oct 13;33(10):1150–70. doi: 10.1089/thy.2023.0281 (PMC10611977; doi:10.1089/thy.2023.0281)
Supplement: Supplemental data [file Suppl_DataS1.docx]

**DISCLOSURES**

Conflict of Interest Disclosures

| First Name | Last Name | Financial Disclosure Form? | Disclosures Noted | Is this relationship  relevant? |
| --- | --- | --- | --- | --- |
|  |  |  |  |  |
| Catherine | Sinclair | Yes | None |  |
| Jennifer | Kuo | Yes | None |  |
| Jung | Baek | Yes | Consultant, Starmed  Consultant, RF Medical | Yes |
| Kathleen | Hands | Yes | None |  |
| Steven | Hodak | Yes | Speaker fees, Sonic Healthcare | No |
| Timothy | Huber | Yes | None |  |
| Iram | Hussain | Yes | Single consulting fee (Veracyte) | No |
| Brian | Lang | Yes | None |  |
| Julia | Noel | Yes | Consultant, Pulse Biosciences | Yes |
| Maria | Papaleontiou | Yes | None |  |
| Kepal | Patel | Yes | None |  |
| Gilles | Russ | Yes | None |  |
| Jonathon | Russell | Yes | Consultant, Baxter Scientific  Consultant, Veracyte | No |
| Stefano | Spiezia | Yes | None |  |
